# Schistosomiasis is associated with incident HIV transmission and death in Zambia

**DOI:** 10.1371/journal.pntd.0006902

**Published:** 2018-12-13

**Authors:** Kristin M. Wall, William Kilembe, Bellington Vwalika, Cecile Dinh, Paul Livingston, Yeuk-Mui Lee, Shabir Lakhi, Debi Boeras, Htee Khu Naw, Ilene Brill, Elwyn Chomba, Tyronza Sharkey, Rachel Parker, Erin Shutes, Amanda Tichacek, W. Evan Secor, Susan Allen

**Affiliations:** 1 Rwanda Zambia HIV Research Group, Department of Pathology & Laboratory Medicine, School of Medicine and Hubert Department of Global Health, Rollins School of Public Health, Emory University, Atlanta, Georgia, United States of America; 2 Department of Epidemiology, Rollins School of Public Health, Laney Graduate School, Emory University, Atlanta, Georgia, United States of America; 3 Department of Gynecology and Obstetrics, School of Medicine, University of Zambia, Lusaka, Zambia; 4 Division of Parasitic Diseases and Malaria, Centers for Disease Control and Prevention, Atlanta, Georgia, United States of America; 5 Department of Internal Medicine, School of Medicine, University of Zambia, Lusaka, Zambia; 6 Department of Epidemiology, Ryals School of Public Health, University of Alabama at Birmingham, Birmingham, Alabama, United States of America; 7 Ministry of Home Affairs, Zambian Ministry of Health, Lusaka, Zambia; 8 School of Medicine, University of Zambia, Lusaka, Zambia; 9 Malaria Team, Global Health Program, Bill & Melinda Gates Foundation, Seattle, Washington, United States of America; London School of Hygiene and Tropical Medicine, UNITED KINGDOM

## Abstract

**Background:**

We examined relationships between schistosome infection, HIV transmission or acquisition, and all-cause death.

**Methods:**

We retrospectively tested baseline sera from a heterosexual HIV-discordant couple cohort in Lusaka, Zambia with follow-up from 1994–2012 in a nested case-control design. Schistosome-specific antibody levels were measured by ELISA. Associations between baseline antibody response to schistosome antigens and incident HIV transmission, acquisition, and all-cause death stratified by gender and HIV status were assessed. In a subset of HIV- women and HIV+ men, we performed immunoblots to evaluate associations between *Schistosoma haematobium* or *Schistosoma mansoni* infection history and HIV incidence.

**Results:**

Of 2,145 individuals, 59% had positive baseline schistosome-specific antibody responses. In HIV+ women and men, baseline schistosome-specific antibodies were associated with HIV transmission to partners (adjusted hazard ratio [aHR] = 1.8, p<0.005 and aHR = 1.4, p<0.05, respectively) and death in HIV+ women (aHR = 2.2, p<0.001). In 250 HIV- women, presence of *S*. *haematobium*-specific antibodies was associated with increased risk of HIV acquisition (aHR = 1.4, p<0.05).

**Conclusion:**

Schistosome infections were associated with increased transmission of HIV from both sexes, acquisition of HIV in women, and increased progression to death in HIV+ women. Establishing effective prevention and treatment strategies for schistosomiasis, including in urban adults, may reduce HIV incidence and death in HIV+ persons living in endemic areas.

## Author summary

This study explored the association between schistosome infections (a disease caused by parasitic flatworms, also known as ‘snail fever’, which is very common throughout sub-Saharan Africa) and human immunodeficiency virus (HIV). We found in Lusaka, the capital of Zambia, that schistosome infections were associated with transmission of HIV from adult men and women, and schistosome infections were also associated with increased HIV acquisition in adult women. We additionally found that schistosome infections were associated with death in HIV+ adult women. Since treatment of schistosome infections with praziquantel is inexpensive, effective, and safe, schistosomiasis prevention and treatment strategies may be a cost-effective way to reduce not only the symptoms associated with the infection, but also new cases of HIV and death among HIV+ persons. Though often viewed as an infection of predominantly rural areas and children, this study highlights that schistosomiasis prevention and treatment efforts are also needed in urban areas and among adults.

## Introduction

Of the more than 200 million persons who have schistosomiasis worldwide, more than 90% live in Africa, and the disease causes tens to hundreds of thousands of deaths annually [1]. Of the parasitic diseases, the health impact of schistosomiasis is second only to malaria [2]. Common species in sub-Saharan Africa are *Schistosoma haematobium* and *Schistosoma mansoni*, which cause urogenital and intestinal schistosomiasis, respectively [2]. Infection is generally established during childhood and is more common among those living in rural areas with frequent freshwater contact [1]. However, urbanization trends have increasingly brought schistosomiasis to municipal areas including Lusaka, the capital of Zambia [3, 4].

Like many of the neglected tropical diseases, preventive chemotherapy using mass drug administration is the primary strategy for controlling schistosomiasis. Though praziquantel treatment is inexpensive, effective, and safe [5], only 14% of adults and 54% of school-aged children estimated to need preventive chemotherapy in 2016 received treatment [6]. Schistosome eggs not passed in urine or stool become lodged in tissues and induce granulomas that cause the pathology associated with schistosomiasis which persists after the death of the egg. For example, in female genital schistosomiasis (FGS), deposition of eggs can occur in the cervix, vagina, and/or vulva putting women at risk for genital epithelial bleeding, sandy patches on the cervix and throughout the genital tract [7, 8], and vaginal inflammation at the histopathological level [9, 10]. These lesions and inflammation can persist long after eggs are deposited, irrespective of treatment [11, 12].

Like other infections that generate a local immune response and/or cause genital lesions (e.g., ulcerative and non-ulcerative sexually transmitted infections (STI) caused by syphilis, herpes simplex virus (HSV), trichomonas, gonorrhea, and chlamydia), schistosomiasis may increase the risk of HIV infection [13, 14]. Cross-sectional studies show associations between urogenital schistosomiasis and HIV prevalence [15]. Though relatively few longitudinal studies have been published, the literature supports the hypothesis that urogenital schistosomiasis is a risk factor for HIV acquisition in HIV- persons and a risk factor for HIV transmission and disease progression in those co-infected with HIV [16–19].

Because it may increase risk of HIV transmission or acquisition, treating schistosomiasis could be a highly cost-effective HIV prevention strategy, and the World Health Organization has called for more studies examining schistosomiasis in HIV endemic countries [15, 20–22]. In Zambia, a country with high prevalence of both HIV (13%, [23]) and schistosomiasis (5–40% [3, 24, 25]), we retrospectively analyzed data from urban adults enrolled in a longitudinal HIV discordant couple cohort to test the hypothesis that there is a relationship between a person having schistosome-specific antibodies (reflecting either active or previous infection, with potential for residual sequelae) and transmitting HIV, acquiring HIV, and all-cause death. We also describe the effect of infecting schistosome species on HIV acquisition among a sub-set of female HIV- and male HIV+ cohort participants.

## Methods

### Ethics statement

The Emory University Office for Human Research Protections-registered Institutional Review Board (IRB) and the Zambian Ethics Committee approved this study. The University of Zambia Biomedical Research Ethics Committee (IORG0000774) is registered with the US Office of Human Research Protection (IRB00001131). Written informed consent was obtained from participants, all of whom were adults. The Centers for Disease Control and Prevention (CDC) also reviewed the protocol; CDC investigators were not considered to be engaged with study participants as they had no direct contact with them and no access to identifying information.

### The cohort

Heterosexual HIV discordant couples (M+F- and M-F+) were enrolled in an open cohort with longitudinal follow-up every three months in Lusaka, Zambia between 1994 and 2012. Participants were identified through couples’ voluntary HIV counseling and testing (CVCT). Study recruitment [26], enrollment, retention and attrition [27], HIV testing and counseling procedures [28, 29], and cohort demographics [30] have been previously published. Briefly, CVCT included group educational sessions, rapid HIV antibody testing, and joint post-test couples’ counseling. HIV serodiscordant heterosexual couples who voluntarily enrolled in the open cohort were provided with free outpatient care including STI testing/treatment and family planning. Couples were censored upon antiretroviral treatment initiation, death of either partner, or relationship dissolution.

Data on demographics (including age, years cohabiting, monthly income, and literacy in Nyanja, the most commonly spoken language in Lusaka Province), family planning, and clinical characteristics (including pregnancy, baseline HIV stage and viral load of HIV+ partners, male partner circumcision status, and STI history) were collected. Past year STI history included self-reported gonorrhea, chlamydia, trichomonas, syphilis, and HSV-2 diagnoses. Viral load was not collected before 1999. Genital abnormalities assessed included discharge or inflammation on visual genital exam (including speculum exam for women). Trichomonas, bacterial vaginosis (BV), and candida infections in women were detected by microscopy of vaginal wet mount swabs (and additional whiff test for BV); genital ulceration (including cervical/vaginal erosion or friability); and syphilis diagnosed via rapid plasma regain (RPR) (BD Macro-Vue, Becton-Dickinson Europe), with *Treponema pallidum* hemagglutination assay confirmation when available [31]. HSV-2 infections were diagnosed by serology testing with the highly sensitive test Focus Diagnostics HerpeSelect 2 ELISA IgG, [32], with repeat testing for indeterminate results.

### Outcomes

HIV incidence was measured via testing of HIV- partners every 1–3 months using rapid antibody tests [29]. When available, plasma from the last antibody negative sample was tested by p24 enzyme-linked immunosorbent assays (ELISA) and RNA polymerase chain reaction (PCR). Based on available data, date of infection was defined as the minimum of: the midpoint between the last negative and first positive antibody test; two weeks prior to a first antigen positive test; or two weeks prior to a first viral load positive/antibody negative test [33]. Date of death was reported by study partners or other family members; >90% of deaths among HIV+ persons were HIV-related [34] and thus death is a proxy for HIV disease progression. Outcomes of interest were relationships between schistosome-specific antibody positivity and time-to incident: HIV acquisition (incident HIV infection in a previously HIV- partner), HIV transmission (onward HIV transmission from an HIV+ index partner), and all-cause death. Our analysis is limited to HIV infections genetically linked to the HIV+ study partner determined via comparison of PCR-amplified conserved nucleotide sequences (*gag*, gp120, gp41, long terminal repeat regions) between partners [35].

### Measurement of schistosomiasis antibodies and species

Blood plasma and serum samples were collected at enrollment from all couples and stored in a repository at Emory University. In 2010, we retrospectively tested plasma samples from individuals enrolled between 1994 and 2009 for antibodies to schistosome soluble worm antigen preparation (SWAP) using a previously described ELISA [36]. All seroconvertors with available samples were included along with a random sample of non seroconvertors in this nested case-control deisgn. ELISA data from each complete discordant pair were not available for some couples. To ensure consistency between plates, a standard 1:3 serial dilution curve was prepared and included on each plate. A 4-parameter curve fitting model was used to assign units based on the standard curve to each unknown sera. The positive cutoff value (25 units) was set at three standard deviations above the average anti-SWAP IgG in serum from egg negative controls from the US and Europe [36]. A positive schistosomiasis result was defined as having a positive SWAP antibody response. A coded list of individuals positive for schistosome-specific antibody was sent to the Director of the Lusaka research site (author WK), and those individuals were offered free praziquantel treatment. Immunoblot testing using species-specific antigens was used to distinguish between *S*. *haematobium* and *S*. *mansoni* antibodies in women and men in a random sample of individuals from the nested case-control study where males were HIV positive and females were negative (M+F-) at baseline [37].

### Statistical data analyses

Descriptive statistics (counts and percentages) described the distribution of schistosomiasis ELISA antibody responses stratified by gender, HIV status, and baseline characteristics, with differences evaluated using Chi-square (or Fisher’s exact) tests for categorical variables and Student’s t-tests for continuous variables. Unadjusted associations between baseline SWAP ELISA results and outcomes of interest were estimated from Cox survival models; crude hazard ratios (cHRs), 95% confidence intervals (CIs), and two-tailed p-values are reported. Adjusted Cox survival models were created and adjusted hazard ratios (aHRs) were calculated adjusting for factors associated (p<0.05) with both the exposure and outcome of interest (the ‘confounding triangle’ method). We also applied a second strategy for adjustment by exploring all subsets of potential confounders to look for meaningful (+/-10%) differences in adjusted hazard ratios. In the subset of individuals in M+F- couples with species-specific immunoblot results, we similarly ran unadjusted and adjusted analyses assessing the association between either *S*. *haematobium* or *S*. *mansoni* and HIV incidence and death. In this subset analysis, we considered the potential for confounding by the other species. All analyses were performed with SAS 9.4 (Cary, NC).

## Results

The average follow-up time for n = 1046 male participants was 801.5 days (standard deviation, SD = 778.1, 2,295.2 total man-years of observation). The average follow-up time for n = 1099 female participants was 816.3 days (SD = 799.4, 2456.3 total woman-years of observation).

In this analysis, 19% of men and 12% of women died during follow-up, 8% of HIV+ men and 10% of HIV+ women initiated ART during follow-up, and 4% of couples separated during follow-up (censoring criteria).

### Distribution of SWAP ELISA results

Of 2,145 individuals tested by SWAP ELISA, 59% were positive for anti-schistosome antibodies at baseline (25% had ELISA levels >70 units, 34% had 25–70 units), and 41% were negative (<25 units). Schistosome-specific antibody levels were higher in men than women (p<0.0001). This difference was driven by a much higher number of males than females with ELISA levels >70 units (31% of men vs. 19% of women) while the frequencies for 25-<50 and 50–70 units were similar for men and women. There were no differences in the distribution of ELISA results when stratifying by sex and HIV status simultaneously (Table 1).

**Table 1:** Baseline schistosome-specific antibody status stratified by sex and HIV status.

	All Women	All Men		HIV+ Women	HIV- Women		HIV+ Men	HIV- Men	
ELISA units	N (%)	N (%)	p-value	N (%)	N (%)	p-value	N (%)	N (%)	p-value
>70	210 (19)	326 (31)	< .0001	111 (19)	99 (20)	0.319	179 (30)	147 (33)	0.320
50–70	95 (9)	95 (9)		52 (9)	43 (9)		53 (9)	42 (9)	
25 –<50	266 (24)	269 (26)		133 (22)	133 (26)		149 (25)	120 (27)	
< 25	528 (48)	356 (34)		300 (50)	228 (45)		218 (36)	138 (31)	
**Total (row %)**	1099 (51)	1046 (49)		596 (28)	503 (23)		599 (28)	447 (21)	

ELISA: enzyme-linked immunosorbent assay

p-values are for comparisons across all four ELISA categories

### Associations between women’s schistosome-specific antibody status and baseline characteristics (Table 2)

**Table 2:** Descriptive statistics and associations between women’s baseline characteristics and schistosome-specific antibody status.

	HIV+ (N = 596)			HIV- (N = 503)		
	Schistosome-specific antibody status			Schistosome-specific antibody status		
	Positive	Negative	p-value	Positive	Negative	p-value
**Demographics**						
**Age (mean, SD)**	28.2 (7.3)	28.3 (6.9)	0.788	27.6 (7.0)	26.6 (6.9)	0.097
**Years cohabiting (mean, SD)**	6.2 (7.2)	5.6 (5.8)	0.222	7.6 (6.5)	7.4 (6.7)	0.740
**Monthly household income (mean USD, SD)**	60.2 (76.5)	71.3 (107.1)	0.147	59.8 (78.6)	67.1 (70.3)	0.282
**Reads Nyanja**			0.628			0.850
Yes, easily (N, %)	68 (23)	74 (25)		50 (19)	40 (18)	
With difficulty/not at all (N, %)	223 (77)	221 (75)		220 (81)	184 (82)	
**Clinical characteristics**						
**Pregnant at baseline**			< .0001			0.033
Yes (N, %)	28 (9)	64 (21)		33 (12)	43 (19)	
No (N, %)	268 (91)	236 (79)		242 (88)	185 (81)	
**HIV stage**			0.001			
Stage I (N, %)	84 (28)	120 (40)				
Stage II (N, %)	84 (28)	93 (31)				
Stage III-IV (N, %)	128 (43)	87 (29)				
**Viral load (mean log**_**10**_ copies/ml, SD)*	4.6 (0.9)	4.4 (0.9)	0.094			
**Past year history of any STI**			0.559			0.844
Yes (N, %)	136 (46)	145 (48)		96 (35)	77 (34)	
No (N, %)	160 (54)	155 (52)		179 (65)	149 (66)	
**Genital conditions (non-ulcerative)**			<0.001			0.822
STI-associated (N, %)	56 (19)	33 (11)		35 (13)	25 (11)	
Non-STI-associated (N, %)	73 (25)	51 (17)		63 (23)	52 (23)	
None (N, %)	167 (56)	216 (72)		177 (64)	151 (66)	
**Genital ulcer**			0.003			0.497
Yes (N, %)	67 (23)	40 (13)		33 (12)	23 (10)	
No (N, %)	229 (77)	260 (87)		242 (88)	205 (90)	
**Baseline schistosome-specific antibody status of male partner**			0.002			0.004
Positive (N, %)	188 (76)	97 (61)		197 (76)	132 (63)	
Negative (N, %)	60 (24)	62 (39)		63 (24)	76 (37)	

SD: standard deviation; STI: sexually transmitted infection; USD: United States Dollar

Genital conditions (non-ulcerative) of STI origin includes: clinical or laboratory diagnosis or treatment of gonorrhea or chlamydia (including presumptive treatment given detection of endocervical discharge) or trichomonas. Genital conditions (non-ulcerative) of non-STI origin includes: reported discharge, dysuria, dyspareunia; observed discharge or inflammation of external or internal genitalia; and/or laboratory diagnosis of candida or bacterial vaginosis (with no indication of an inflammatory STI). Genital ulcer includes: observed or reported ulcers and/or baseline positive rapid plasma regain status greater than a titer of 1:2. Genital conditions (non-ulcerative) categories are based on Wall et al [38].

*Not collected before 1999 (samples available for N = 410 HIV+ women).

Women with positive schistosome-specific ELISA results were less likely to be pregnant at baseline (p<0.0001). Positive schistosome-specific ELISA results were also associated with signs and symptoms of genital conditions (non-ulcerative) of STI or non-STI etiologies (p<0.001) and genital ulcers (p = 0.003) in HIV+ women. HIV+ women who were positive for schistosome-specific antibodies were more likely to be at an advanced HIV disease stage III-IV (p = 0.001). In addition, women who were positive for schistosome-specific antibodies were more likely to have a male partner that was also schistosome-specific antibody positive (p<0.01). However, a woman’s age, duration of cohabitation, household income, literacy, and viral load were not statistically significantly associated with schistosome-specific ELISA status. Similarly, number of prior pregnancies, baseline HSV-2 antibody status, or baseline RPR results were not associated with schistosome-specific ELISA status (data not tabled). In the subset of women with information on fertility intentions, tribal/linguistic group, or where they lived prior to age 16 (which was only collected after 2002), there was also no association with schistosome-specific ELISA status (data not tabled). Most data were missing for fertility intentions (72%), tribal/linguistic group (69%), or where they lived prior to age 16 (81%).

### Associations between men’s schistosome-specific antibody status and baseline characteristics (Table 3)

**Table 3:** Descriptive statistics and associations between men’s baseline characteristics and schistosome-specific antibody status.

	HIV+ (N = 599)			HIV- (N = 447)		
	Schistosome-specific antibody status			Schistosome-specific antibody status		
	Positive	Negative	p-value	Positive	Negative	p-value
**Demographics**						
**Age (mean, SD)**	34.3 (7.6)	34.2 (7.9)	0.955	35.1 (9.2)	33.5 (8.7)	0.073
**Years cohabiting (mean, SD)**	7.6 (6.6)	7.2 (6.1)	0.458	5.7 (7.0)	5.3 (4.9)	0.637
**Monthly household income (mean USD, SD)**	61.0 (79.2)	72.1 (82.8)	0.109	60.7 (78.5)	69.5 (117.6)	0.352
**Reads Nyanja**			0.029			0.432
Yes, easily (N, %)	181 (49)	81 (40)		124 (42)	58 (46)	
With difficulty/not at all (N, %)	187 (51)	123 (60)		172 (58)	68 (54)	
**Clinical characteristics**						
**HIV stage**			0.912			
Stage I (N, %)	82 (22)	50 (23)				
Stage II (N, %)	145 (38)	77 (35)				
Stage III-IV (N, %)	154 (40)	91 (42)				
**Viral load (mean log**_**10**_ copies/ml, SD)*	5.0 (0.8)	4.8 (0.9)	0.025			
**Circumcised**			0.004			0.373
Yes (N, %)	42 (11)	9 (4)		48 (16)	17 (12)	
No (N, %)	339 (89)	209 (96)		261 (84)	121 (88)	
**Past year history of any STI**			0.311			0.442
Yes (N, %)	178 (47)	111 (51)		107 (35)	53 (38)	
No (N, %)	202 (53)	106 (49)		202 (65)	85 (62)	
**Genital conditions (non-ulcerative)**			0.358			0.062
STI (N, %)	17 (4)	5 (2)		12 (4)	2 (1)	
Non-STI (N, %)	7 (2)	3 (1)		23 (7)	4 (3)	
No (N, %)	357 (94)	210 (96)		274 (89)	132 (96)	
**Genital ulcer**			0.532			0.067
Yes (N, %)	67 (18)	34 (16)		35 (11)	8 (6)	
No (N, %)	314 (82)	184 (84)		274 (89)	130 (94)	
**Baseline schistosome-specific antibody status of female partner**			0.004			0.002
Positive (N, %)	197 (60)	63 (45)		188 (66)	60 (49)	
Negative (N, %)	132 (40)	76 (55)		97 (34)	62 (51)	

SD: standard deviation; STI: sexually transmitted infection; USD: United States Dollar

Genital conditions (non-ulcerative) of STI origin includes: clinical or laboratory diagnosis or treatment of gonorrhea or chlamydia (including presumptive treatment given detection of urethral discharge). Genital conditions (non-ulcerative) of non-STI origin includes: reported discharge, dysuria, dyspareunia; and/or observed discharge or inflammation of external genitalia (with no indication of an inflammatory STI). Genital ulcer includes: observed or reported ulcers and/or baseline positive rapid plasma regain status greater than a titer of 1:2. Genital conditions (non-ulcerative) categories are based on Wall et al [38].

*Not collected before 1999 (samples available for N = 417 HIV+ men).

Unlike in women, presence of schistosome-specific antibodies in HIV+ men were not related to HIV disease stage but viral loads were higher in men with positive schistosome-specific antibody status (p = 0.025). Interestingly, positive schistosome-specific antibody status was associated with an increased likelihood of being circumcised among HIV+ men (p = 0.004) and increased literacy (p = 0.029). Among HIV- men, genital conditions (non-ulcerative) (p = 0.062) and genital ulcer (p = 0.067) were not statistically significantly associated with a positive schistosome-specific antibody result. In addition, men who were positive for schistosome-specific antibodies were more likely to have a female partner that was also schistosome-specific antibody positive (p<0.01). Men’s age, duration of cohabitation, household income, literacy, and reported history of STI in the last year were not associated with schistosome-specific antibody responses. Nor was baseline HSV-2 antibody status, baseline RPR results, or (in a subset of men with the following information which was only collected after 2002) fertility intentions, tribal/linguistic group, or where they lived prior to age 16 associated with schistosome-specific antibody status (data not tabled). Most data were missing for fertility intentions (75%), tribal/linguistic group (72%), or where they lived prior to age 16 (83%).

### Associations between women’s baseline schistosome-specific antibody status and HIV transmission and acquisition (Fig 1, S1 Table)

**Fig 1:**
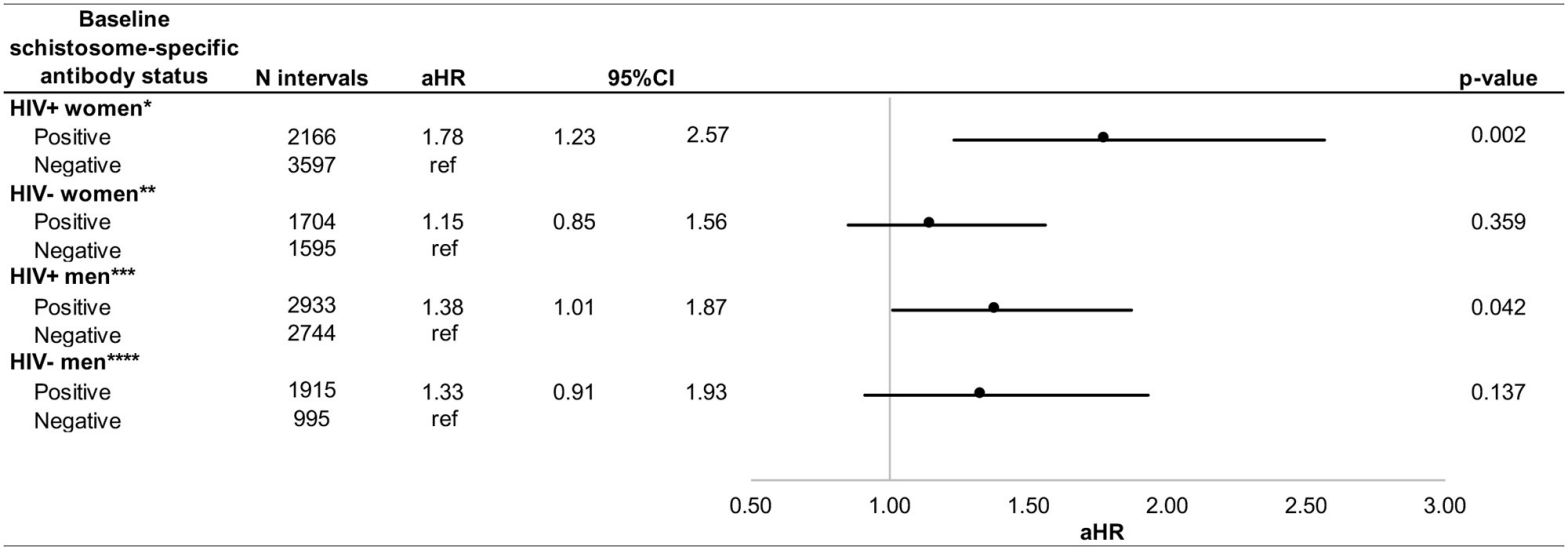
Adjusted associations between women's and men's baseline schistosome-specific antibody status and HIV transmission and acquisition. CI: confidence interval; aHR: adjusted hazard ratio *Controlling for factors associated with both the exposure and outcome of interest: Genital conditions (non-ulcerative) of woman, genital ulcer of woman **Controlling for factors associated with both the exposure and outcome of interest: Male partner's baseline schistosome-specific antibody status ***Controlling for factors associated with both the exposure and outcome of interest: Viral load of man ****Controlling for factors associated with both the exposure and outcome of interest: Female partner's baseline schistosome-specific antibody status.

We observed N = 70/296 and N = 70/300 linked HIV transmission outcomes for baseline schistosome-specific antibody positive and negative HIV+ women, respectively. We observed N = 92/275 and N = 97/228 linked HIV acquisition outcomes for baseline schistosome-specific antibody positive and negative HIV- women, respectively. In unadjusted analyses, HIV+ women positive for schistosome-specific antibodies at baseline had a shorter time-to-HIV transmission to HIV- male partners (cHR = 1.8, p = 0.002). When multivariate analyses were performed controlling for genital conditions (non-ulcerative) and genital ulceration of the woman, positive baseline schistosome-specific antibody in HIV+ women remained associated with shorter time-to-HIV transmission to HIV- male partners (aHR = 1.8, p = 0.002). When applying a different strategy for adjustment (exploring at all subsets of potential confounders to look for meaningful (+/-10%) differences in adjusted hazard ratios), we arrived at the same set of confounders as when using the present (‘confounding triangle’) method. Because of the nested case-control design, proportions of outcomes should not be calculated/interpreted as risk estimates.

### Associations between men’s baseline schistosome-specific antibody status and HIV transmission and acquisition (Fig 1, S2 Table)

We observed N = 112/381 and N = 78/218 HIV transmission outcomes for baseline schistosome-specific antibody positive and negative HIV+ men, respectively. We observed N = 93/309 and N = 53/138 HIV acquisition outcomes for baseline schistosome-specific antibody positive and negative HIV- men, respectively. In unadjusted analyses, HIV+ men positive for schistosome-specific antibodies at baseline had a shorter time-to-HIV transmission to HIV- female partners (cHR = 1.4, p = 0.016). When multivariate analyses were performed controlling for men’s viral load, positive baseline schistosome-specific antibody in HIV+ men remained associated with shorter time-to-HIV transmission to HIV- female partners (aHR = 1.4, p = 0.042). When applying a different strategy for adjustment (exploring at all subsets of potential confounders to look for meaningful (+/-10%) differences in adjusted hazard ratios), we arrived at the same set of confounders as when using the present (‘confounding triangle’) method. Because of the nested case-control design, proportions of outcomes should not be calculated/interpreted as risk estimates.

### Associations between women’s baseline schistosome-specific antibody status and death (Fig 2, S3 Table)

**Fig 2:**
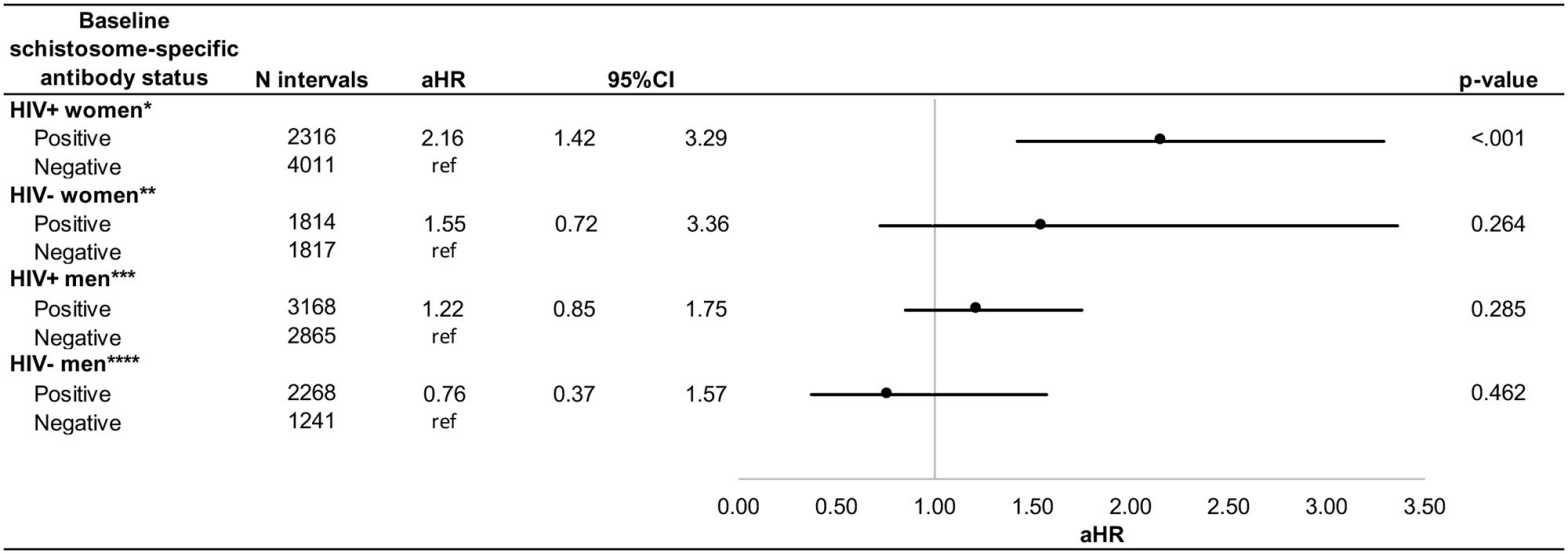
Adjusted associations between women's and men's baseline schistosome-specific antibody status and death. CI: confidence interval; aHR: adjusted hazard ratio *Controlling for factors associated with both the exposure and outcome of interest: HIV stage of woman **Unadjusted model ***Controlling for factors associated with both the exposure and outcome of interest: Viral load of man ****Controlling for factors associated with both the exposure and outcome of interest: Female partner's baseline schistosome-specific antibody status.

We observed N = 65/296 and N = 44/300 death outcomes for baseline schistosome-specific antibody positive and negative HIV+ women, respectively. We observed N = 17/275 and N = 11/228 death outcomes for baseline schistosome-specific antibody positive and negative HIV- women, respectively. In unadjusted analyses, HIV+ women positive for schistosome-specific antibodies at baseline had a shorter time-to-women’s death (cHR = 2.3, p<0.0001). When multivariate analyses were performed controlling for HIV stage of the woman, positive baseline schistosome-specific antibody in HIV+ women remained associated with shorter time-to-women’s death (aHR = 2.2, p<0.001). When applying a different strategy for adjustment (exploring at all subsets of potential confounders to look for meaningful (+/-10%) differences in adjusted hazard ratios), we arrived at the same set of confounders as when using the present (‘confounding triangle’) method. When adjusting for viral load of the HIV+ woman (as was done in the model for men, below), the point estimate is slightly tempered but still significant (aHR = 1.97; 95%CI:1.19–3.25, p-value = 0.008) (data not tabeled).

### Associations between men’s baseline schistosome-specific antibody status and death (Fig 2, S4 Table)

We observed N = 110/381 and N = 56/218 death outcomes for baseline schistosome-specific antibody positive and negative HIV+ men, respectively. We observed N = 21/309 and N = 13/138 death outcomes for baseline schistosome-specific antibody positive and negative HIV- men, respectively. In unadjusted analyses, HIV+ men positive for schistosome-specific antibodies at baseline were associated with decreased time-to-men’s death (cHR = 1.6, p = 0.008). Baseline schistosome-specific antibody levels were not significantly associated with time-to-HIV+ men’s death once adjusting for men’s viral load. However, viral load was not collected before 1999, thus only 126 of 166 death events were modeled when controlling for viral load. When applying a different strategy for adjustment (exploring at all subsets of potential confounders to look for meaningful (+/-10%) differences in adjusted hazard ratios), we arrived at the same set of confounders as when using the present (‘confounding triangle’) method.

### Associations between schistosomiasis species-specific immunoblot results and HIV acquisition and transmission (Table 4)

**Table 4:** Unadjusted and adjusted associations between schistosomiasis species-specific immunoblot results and HIV acquisition and transmission.

**Women's results**	**Woman HIV-**									
	**Linked HIV acquisitions**	**Non-HIV acquiring**	**cHR**	**95%CI**		**p-value**	aHR*	**95%CI**		**p-value**
***S*. *haematobium***										
Positive (N intervals, %)	63 (30)	391 (26)	1.44	1.05	1.96	0.022	1.40	1.03	1.92	0.034
Negative (N intervals, %)	144 (70)	1108 (74)	ref				ref			
***S*. *mansoni***										
Positive (N intervals, %)	40 (19)	265 (18)	1.30	0.91	1.87	0.147	1.33	0.93	1.91	0.121
Negative (N intervals, %)	167 (81)	1234 (82)	ref				ref			
**Men's results**	**Man HIV +**									
	**Linked HIV trans-missions**	**Non-HIV transmitting**	**cHR**	**95%CI**		**p-value**	aHR**	**95%CI**		**p-value**
***S*. *haematobium***										
Positive (N intervals, %)	64 (33)	492 (35)	0.94	0.69	1.27	0.676	0.88	0.65	1.20	0.422
Negative (N intervals, %)	132 (67)	909 (65)	ref				ref			
***S*. *mansoni***										
Positive (N intervals, %)	44 (22)	197 (14)	1.37	0.97	1.93	0.071	1.33	0.94	1.88	0.111
Negative (N intervals, %)	152 (78)	1204 (86)	ref				ref			

cHR: crude hazard ratio; CI: confidence interval; aHR: adjusted hazard ratio

p-values are two-tailed

*Controlling for genital conditions (non-ulcerative) of woman, genital ulcer of woman

**Controlling for viral load of the man

Among 250 HIV- women and 239 HIV+ men, we observed N = 63/76 and N = 144/174 HIV acquisition outcomes for *S*. *haematobium* positive and negative HIV- women, respectively. We observed N = 40/46 and N = 167/204 HIV acquisition outcomes for *S*. *mansoni* positive and negative HIV- women, respectively. We observed N = 64/80 and N = 132/159 HIV transmission outcomes for *S*. *haematobium* positive and negative HIV+ men, respectively. We observed N = 44/54 and N = 152/185 HIV transmission outcomes for *S*. *mansoni* positive and negative HIV+ men, respectively. In M+F- HIV discordant couples, species-specific schistosome antibody response were evaluated by species-specific immunoblot results. *S*. *haematobium*-specific antibodies (aHR = 1.4, p = 0.034) significantly increased the risk HIV acquisition in women, while *S*. *mansoni*-specific antibodies also increased risk of HIV acquisition, though not significantly (aHR = 1.3, p = 0.12). The schistosome species infecting men did not significantly influence the likelihood of viral transmission to their HIV- partners. Notably, we had very limited power to detect the difference between *S*. *mansoni* status and HIV acquisition in HIV- women (3%) or *S*. *mansoni* status and HIV transmission from HIV+ men (19%). When applying a different strategy for adjustment (exploring at all subsets of potential confounders to look for meaningful (+/-10%) differences in adjusted hazard ratios), we arrived at the same set of confounders as when using the present (‘confounding triangle’) method. Because of the nested case-control design, proportions of outcomes should not be calculated/interpreted as risk estimates.

## Discussion

Our findings indicate a high prevalence of antibodies to schistosomes that was associated with onward HIV transmission from HIV+ men and women and earlier death in HIV+ women. *S*. *haematobium* specific immunoblot reactivity was associated (p = 0.034) with increased HIV acquisition risk in HIV- women.

In this study, 59% of participants were positive for schistosome-specific antibodies, indicating an unexpectedly high prevalence of current or past infection in our urban population. Past infection can lead to persistent residual sequelae, which may increase HIV risk irrespective of treatment [11, 12]. Men had higher ELISA values at baseline than women, possibly explained by the finding that more men than women had lived in a rural area prior to age 16. Rural areas in Zambia have a higher prevalence of schistosomiasis, and most infections are acquired during youth [39]. The high observed prevalence of schistosome-specific antibodies in this urban population necessitates a shift in thinking of schistosomiasis as only a disease of children and rural areas. More research is needed to examine urbanization and migration in schistosomiasis transmission, especially in endemic countries. For example, individuals in Lusaka without access to effective water delivery systems may rely on both piped and environmental water sources, which might increase their risk of acquiring schistosome infection [25].

Past or baseline schistosome infection in HIV+ partners was significantly associated with onward HIV transmission to HIV- partners. A possible explanation is increased viral load in the HIV+ partners. In co-infected HIV+ men, schistosome egg excretion in semen may be accompanied by increases in lymphocytes, eosinophils [40], and other cells associated with HIV replication. Furthermore, HIV binding receptors (CCR5 and CXC4) are denser on CD4 T-cell surfaces in men with intestinal schistosomiasis compared to uninfected individuals and those treated with praziquantel [41]. In co-infected HIV+ women, genital schistosomiasis may lead to inflammatory genital lesions that increase transmissibility of HIV [13].

We also observed that *S*. *haematobium* (but not *S*. *mansoni*) infection as determined by immunoblot was associated increased risk of HIV acquisition by HIV- women in discordant relationships. This finding is consistent with the association of urogenital schistosomiasis and FGS and is similar, though of lesser magnitude, to results from studies in rural Zimbabwe [13] and Tanzania [14, 42] that showed schistosomiasis was associated with a 2–3 fold increased HIV risk in women. However, prior studies did not rely on antibody status indicating past or current infection but rather active infection, limiting direct comparisons with our study. FGS may increase susceptibility to HIV due to cervical lesions reducing the integrity of the genital epithelial barrier [7, 10, 13, 43] or recruitment of CD4+ lymphocytes, macrophages, and Langerhans giant cells [10] to the genital tract, thus increasing the probability of HIV infection [9]. However, most previous research regarding FGS and inflammation has been at the histopathological level without biopsy [9, 10].

A previous randomized controlled trial detected no effect of active schistosome infection on time-to-a composite indicator of HIV disease progression (first occurrence of a CD4 count of <350 cells/ml, first reported use of antiretroviral treatment, and non-traumatic death) [44]. Conversely, a longitudinal study in Tanzania found that individuals with active schistosome infection at the time of their HIV seroconversion had slower HIV disease progression (as indicated by CD4 count of <350 cells/ml or death) [45]. The authors suggest that this unexpected finding indicated complicated interactions between long-term HIV immunological changes and schistosome co-infections, and that additional studies are needed. Furthermore, a 2016 Cochrane Review found scant, “low quality” evidence that treating helminth infections has beneficial effects on slowing HIV disease progression [46]. By contrast, our study is the first to investigate schistosome-specific antibody status, reflecting either past or current infection, as a factor associated with mortality in HIV+ individuals. Interestingly, we also observed an association between schistosome-specific antibody responses and increased baseline HIV stage in women and viral load in men. More research will be needed to confirm these findings.

Women who were not pregnant at baseline were more likely to be schistosome-specific antibody positive. Previous research, including case reports, ecological studies, descriptive series, and geographical mapping, has indicated an association between *S*. *haematobium* infection and decreased fertility in sub-Saharan Africa [47, 48]. In a cross-sectional interview study in Kenya, documented treatment for childhood urogenital *S*. *haematobium* among women was associated with decreased fertility in adulthood [49], further highlighting the importance of primary prevention for urogenital schistosomiasis and early treatment.

We also found that schistosome-specific antibody positive responses were associated with male circumcision (significant association for HIV+ men, with a trend for HIV- men). To explain this finding, we evaluated circumcision by education, occupation, income, tribal/linguistic group, and whether the man lived in an urban versus rural area before the age of 16. However, none of these factors attenuated the association between circumcision and ELISA results (data not tabled). This finding warrants further exploration.

Given the high prevalence of a history of schistosomiasis in our study, the associated risk of HIV transmission and death among HIV+ persons positive for schistosome-specific antibody, and other studies indicating an increased risk of HIV transmission/acquisition due to schistosome infection, our findings underscore the importance of schistosomiasis treatment and prevention. Further, we argue for integration of routine parasitological testing and treatment in HIV programs. Treating schistosomiasis has been proposed as a cost-effective addition to HIV prevention and treatment programs, and may contribute to slowing the spread of HIV while reducing schistosomiasis-associated morbidity [21]. Praziquantel treatment for schistosomiasis is safe, including in pregnant women [5, 16], has no reported widespread drug resistance, has only moderate side-effects, and can be dispensed via community-wide mass administration [20]. Additionally, praziquantel may attenuate HIV replication by decreasing systemic inflammation [50] and slow HIV disease progression [19, 46, 50, 51]. HIV+ individuals with delayed schistosomiasis treatment have increased viral loads and lower CD4 T-cell counts compared to those who received early treatment [19], and antihelminthic drugs may act to reduce viral load and increase CD4 levels [51]. In resource-limited countries such as Zambia, more efforts are needed to train health care providers, including HIV practitioners, to detect schistosomiasis and administer praziquantel. It will also be important for policy makers to consider the cost-effectiveness of new methods to detect FGS morbidity versus long-term programmatic benefits.

Our study has limitations. Though ELISA tests can be highly specific and robust laboratory measures of antibodies to schistosome infection [52] and the use of immunoblots to detect schistosomiasis species has been validated for *S*. *mansoni* [53, 54] and S. *haematobium* [55], it is possible that some of the participants were misclassified. In a study in Western Kenya, the SWAP ELISA had a sensitivity of 92% and specificity of 57% when compared to fecal egg microscopy, which only detects active infection. The lower specificity of the ELISA is likely associated with identifying people who were previously infected and/or the relatively lower sensitivity of the parasitological method [36]. ELISA cutoff values would have been more compelling if we had known negative controls from Zambian samples. Furthermore, it would have been informative if we had performed sensitive diagnostic procedures, including antigen detection or schistosomiasis PCR, to delineate active infections. Nevertheless, our data support the growing appreciation that the sequelae of schistosomiasis persist beyond the period of active infection. Colposcopy or cervical biopsy to confirm schistosome eggs in urogenital tissue would have enabled definitive diagnosis of FGS although the latter approach has ethical concerns due to creating a genital tract wound in a population of HIV positive or HIV serodiscordant couples.

Schistosomal antibodies were only measured at baseline (along with several other covariates such as viral load), due to funding constraints, and time-varying measures would have been informative to explore how titers changed over time. Type-specific immunoblot testing was only done in women and men who were in M+F- partnerships (primarily to test the hypothesis that female schistosomiasis infection was associated with risk of HIV acquisition in women), and it would have been informative to have species-specific immunoblot data for men and women in M-F+ partnerships. We do not know whether the significantly elevated viral loads (in schistosome-specific antibody positive men) and trend towards higher viral loads (in schistosome-specific antibody positive women) reflect viral loads early in HIV infection or after several years because we do not know time of infection of the HIV+ index partner. The imperfect specificity of the Focus HSV-2 test (sensitivity of 99.5% and specificity of 70.2% in HIV+ and HIV- participants in urban Uganda [56]) is a limitation, and unfortunately due to funding changes, not all RPR results were confirmed by *Treponema pallidum* hemagglutination assay. It is unknown if these sources of potential bias would lead to unmeasured confounding by HSV-2 or RPR status in our analysis. We also lack information on some potentially important covariates, including exposures to environmental water, poor sanitation, prior use of praziquantel, and past socioeconomic status. Finally, we did not have sufficient sample size to look at antiretroviral treatment initiation outcomes (another proxy of HIV disease progression) since this intervention only began in 2007.

Expanded identification and treatment of schistosomiasis are warranted in endemic countries such as Zambia, including in adults in urban areas. In addition to reducing morbidity and mortality associated with schistosomiasis, our findings suggest such efforts might also contribute to prevention of onward HIV transmission and disease progression in HIV+ men and women. As such, the strategy of preventive chemotherapy may have benefits not just for schistosomiasis, but also for HIV. Given the relatively few quantitative studies to date, additional research on schistosomiasis and HIV transmission, acquisition, and disease progression in both men and women, including the potential effects of former infections, are warranted.

## Supporting information

S1 Table Unadjusted and adjusted associations between women's baseline schistosome-specific antibody status and HIV transmission and acquisition. (DOCX)

S2 Table Unadjusted and adjusted associations between men's baseline schistosome-specific antibody status and HIV transmission and acquisition. (DOCX)

S3 Table Unadjusted and adjusted associations between women's baseline schistosome-specific antibody status and death. (DOCX)

S4 Table Unadjusted and adjusted associations between men's baseline schistosome-specific antibody status and death. (DOCX)
