# Supplementary material for: Schistosomiasis is associated with incident HIV transmission and death in Zambia
Source: PLoS Negl Trop Dis. 2018 Dec 13;12(12):e0006902. doi: 10.1371/journal.pntd.0006902 (PMC6292564; doi:10.1371/journal.pntd.0006902)
Supplement: S1 Table — (DOCX) [file pntd.0006902.s001.docx]

**S1 Table. Unadjusted and adjusted associations between women's baseline schistosome-specific antibody status and HIV transmission and acquisition**

| S1 Table Legend:  *Controlling for factors associated with both the exposure and outcome of interest: Genital conditions (non-ulcerative) of woman, genital ulcer of woman | | | |
| --- | --- | --- | --- |
| **Controlling for factors associated with both the exposure and outcome of interest: Male partner's baseline schistosome-specific antibody status | |  |  |
| cHR: crude hazard ratio; CI: confidence interval; aHR: adjusted hazard ratio |  |  |  |
