# Supplementary material for: Schistosomiasis is associated with incident HIV transmission and death in Zambia
Source: PLoS Negl Trop Dis. 2018 Dec 13;12(12):e0006902. doi: 10.1371/journal.pntd.0006902 (PMC6292564; doi:10.1371/journal.pntd.0006902)
Supplement: S2 Table — (DOCX) [file pntd.0006902.s002.docx]

**S2 Table. Unadjusted and adjusted associations between men's baseline schistosome-specific antibody status and HIV transmission and acquisition**

| S2 Table Legend:  *Controlling for factors associated with both the exposure and outcome of interest: Viral load of man | | | |
| --- | --- | --- | --- |
| **Controlling for factors associated with both the exposure and outcome of interest: Female partner's baseline schistosome-specific antibody status | |  |  |
| cHR: crude hazard ratio; CI: confidence interval; aHR: adjusted hazard ratio |  |  |  |
