# Supplementary material for: Schistosomiasis is associated with incident HIV transmission and death in Zambia
Source: PLoS Negl Trop Dis. 2018 Dec 13;12(12):e0006902. doi: 10.1371/journal.pntd.0006902 (PMC6292564; doi:10.1371/journal.pntd.0006902)
Supplement: S3 Table — (DOCX) [file pntd.0006902.s003.docx]

| S3 Table. Unadjusted and adjusted associations between women's baseline schistosome-specific antibody status and death | | | | | | | | | | |  |  |  |  |
| --- | --- | --- | --- | --- | --- | --- | --- | --- | --- | --- | --- | --- | --- | --- |
| **Women's baseline schistosome-specific antibody status** | **Woman HIV+** | | | | | | | | | |  |  |  |  |
|  | **Death of woman** | **No death of woman** | **cHR** | **95%CI** | | **p-value** | **aHR*** | **95%CI** | | **p-value** |  |  |  |  |
| Positive (N intervals, %) | 65 (60) | 2251 (36) | 2.27 | 1.51 | 3.42 | <.0001 | 2.16 | 1.42 | 3.29 | <.001 |  |  |  |  |
| Negative (N intervals, %) | 44 (40) | 3967 (64) | ref |  |  |  | ref |  |  |  |  |  |  |  |
| **Women's baseline schistosome-specific antibody status** | **Woman HIV-** | | | | | | | | | |  |  |  |  |
|  | **Death of woman** | **No death of woman** | **cHR** | **95%CI** | | **p-value** |  |  | |  |  |  |  |  |
| Positive (N intervals, %) | 17 (61) | 1797 (50) | 1.55 | 0.72 | 3.36 | 0.264 | *Same as unadjusted model* | | | |  |  |  |  |
| Negative (N intervals, %) | 11 (39) | 1806 (50) | ref |  |  |  |  |  |  |  |  |  |  |  |
| *Controlling for factors associated with both the exposure and outcome of interest: HIV stage of woman | | | | | | | |  |  |  |  | |  |  |
| cHR: crude hazard ratio; CI: confidence interval; aHR: adjusted hazard ratio | | |  |  |  |  |  |  |  |  |  |  |  |  |
