# Supplementary material for: Schistosomiasis is associated with incident HIV transmission and death in Zambia
Source: PLoS Negl Trop Dis. 2018 Dec 13;12(12):e0006902. doi: 10.1371/journal.pntd.0006902 (PMC6292564; doi:10.1371/journal.pntd.0006902)
Supplement: S4 Table — (DOCX) [file pntd.0006902.s004.docx]

| S4 Table. Unadjusted and adjusted associations between men's baseline schistosome-specific antibody status and death | | | | | | | | | | |  |  |  |  |
| --- | --- | --- | --- | --- | --- | --- | --- | --- | --- | --- | --- | --- | --- | --- |
| **Men's baseline schistosome-specific antibody status** | **Man HIV+** | | | | | | | | | |  |  |  |  |
|  | **Death of man** | **No death of man** | **cHR** | **95%CI** | | **p-value** | **aHR*** | **95%CI** | | **p-value** |  |  |  |  |
| Positive (N intervals, %) | 110 (66) | 3058 (52) | 1.57 | 1.13 | 2.18 | 0.008 | 1.22 | 0.85 | 1.75 | 0.285 |  |  |  |  |
| Negative (N intervals, %) | 56 (34) | 2809 (48) | ref |  |  |  | ref |  |  |  |  |  |  |  |
| **Men's baseline schistosome-specific antibody status** | **Man HIV-** | | | | | | | | | |  |  |  |  |
|  | **Death of man** | **No death of man** | **cHR** | **95%CI** | | **p-value** | **aHR**** | **95%CI** | | **p-value** |  |  |  |  |
| Positive (N intervals, %) | 21 (62) | 2247 (65) | 0.89 | 0.45 | 1.80 | 0.754 | 0.76 | 0.37 1.57 | | 0.462 |  |  |  |  |
| Negative (N intervals, %) | 13 (38) | 1228 (35) | ref |  |  |  | ref |  |  |  |  |  |  |  |
| *Controlling for factors associated with both the exposure and outcome of interest: Viral load of man | | | | | | | |  |  |  |  | |  |  |
| **Controlling for factors associated with both the exposure and outcome of interest: Female partner's baseline schistosome-specific antibody status  cHR: crude hazard ratio; CI: confidence interval; aHR: adjusted hazard ratio | | | | | | | | | | |  |  |  |  |
